# Fishery Wastes as a Yet Undiscovered Treasure from the Sea: Biomolecules Sources, Extraction Methods and Valorization

**DOI:** 10.3390/md18120622

**Published:** 2020-12-07

**Authors:** Gabriella Caruso, Rosanna Floris, Claudio Serangeli, Luisa Di Paola

**Affiliations:** 1 Institute of Polar Sciences, National Research Council, 98122 Messina, Italy; 2 AGRIS-Sardegna, Servizio Ricerca Prodotti Ittici, Bonassai, 07100 Sassari, Italy; rfloris@agrisricerca.it; 3 AGC98 Srl, 00197 Rome, Italy; agc@agc98.net; 4 Unit of Chemical-Physics Fundamentals in Chemical Engineering, Department of Engineering, Università Campus Bio-Medico di Roma, 00128 Rome, Italy; l.dipaola@unicampus.it

**Keywords:** fishery by-catch, processing by-products, bioactive metabolites, enzymes, probiotics, antibiotics, bioprospecting, circular economy, biorefinery

## Abstract

The search for new biological sources of commercial value is a major goal for the sustainable management of natural resources. The huge amount of fishery by-catch or processing by-products continuously produced needs to be managed to avoid environmental problems and keep resource sustainability. Fishery by-products can represent an interesting source of high added value bioactive compounds, such as proteins, carbohydrates, collagen, polyunsaturated fatty acids, chitin, polyphenolic constituents, carotenoids, vitamins, alkaloids, tocopherols, tocotrienols, toxins; nevertheless, their biotechnological potential is still largely underutilized. Depending on their structural and functional characteristics, marine-derived biomolecules can find several applications in food industry, agriculture, biotechnological (chemical, industrial or environmental) fields. Fish internal organs are a rich and underexplored source of bioactive compounds; the fish gut microbiota biosynthesizes essential or short-chain fatty acids, vitamins, minerals or enzymes and is also a source of probiotic candidates, in turn producing bioactive compounds with antibiotic and biosurfactant/bioemulsifier activities. Chemical, enzymatic and/or microbial processing of fishery by-catch or processing by-products allows the production of different valuable bioactive compounds; to date, however, the lack of cost-effective extraction strategies so far has prevented their exploitation on a large scale. Standardization and optimization of extraction procedures are urgently required, as processing conditions can affect the qualitative and quantitative properties of these biomolecules. Valorization routes for such raw materials can provide a great additional value for companies involved in the field of bioprospecting. The present review aims at collecting current knowledge on fishery by-catch or by-products, exploring the valorization of their active biomolecules, in application of the circular economy paradigm applied to the fishery field. It will address specific issues from a biorefinery perspective: (i) fish tissues and organs as potential sources of metabolites, antibiotics and probiotics; (ii) screening for bioactive compounds; (iii) extraction processes and innovative technologies for purification and chemical characterization; (iv) energy production technologies for the exhausted biomass. We provide a general perspective on the techno-economic feasibility and the environmental footprint of the production process, as well as on the definition of legal constraints for the new products production and commercial use.

## 1. The Problem of Fishery By-Catch or Processing By-Products

Fishery resources represent a significant sector of our economy; according to recent estimates [1] more than 170 million metric tons of fish (including capture and aquaculture), shellfish and others are produced each year. The fish processing industry produces a huge amount (ranging from over 25% to 70%) of by-products, made up of viscera, heads, scales, bones, etc., that represent a significant waste, worthy to be valorized. According to Arvanitoyannis and Kassaveti [2], there are around 26% of total food stocks (156.4 million tons) which have not found any application yet, accounting globally to 40.66 million tons of wastes. The latest estimates by the FAO [3] report that global catches, including aquaculture, marine and inland fisheries account for 178.5 million tons of live weight; of which 156.4 tons are destined to human consumption and 22.2 for non-food uses. The non-food uses includes fishmeal and fish oil production, generating practically no waste. As a matter of fact, the residual solid from fish oil extraction is used to produce fishmeal. Reasonably, for logistic issues, only by-products coming from direct processing can be gathered and eventually processed; according to FAO data, around 60% of catches come to processing for food uses (107 million tons), thus resulting in 27.85 million tons of accessible discards on a global scale.

Another relevant source of fishery by-products results from the introduction of the Landing Obligation of the European Common Fisheries Policy [4], that since 2019 proscribed to all commercial fisheries to preserve all caught species that do not reach a minimum legal size or that are subjected to quota, including those that are considered to have a low commercial value or that are damaged specimens. Regarding the Italian marine fishery, in the Mediterranean Sea it has been estimated that the discarded fraction (7% of gross catch) corresponded to about 95% of industrial fisheries in the period between 1950 and 2010 and that the discarding practice has gradually increased over the past 70 years [5], highlighting that fishery discards derived from trawl fleets are an undiscovered treasure.

The problem of fishery discards (including both fishery by-catch and processing by-products) has therefore been recognized as an emerging issue [6,7,8], stimulating the search for possible solutions. Indeed, fishery discards involve management issues, as they are highly perishable due to the high content of polyunsaturated lipids and proteolytic enzymes, and they must be disposed as certified by-products; however, the cost of their disposal strongly affects the economic balance of fishery and fish-processing companies. Consequently, the use of fishery discards as a secondary raw material could be proposed as a resource-efficient approach to manage this problem, avoiding possible environmental impacts and contributing at the same time to keep fisheries sustainability [9,10,11,12]. On the other hand, marine organisms and their by-products have been recognized as an extraordinary source of new bioactive metabolites [13,14,15,16,17,18,19,20,21]. The huge biodiversity of the living organisms inhabiting marine habitats, that accounts for approximately one-half of the total global biodiversity, results in a wide range of structurally diverse bio-functional components [22].

The main issues associated to the problem of fishery by-catch and processing by-products are summarized in Table 1.

Many compounds of marine origin are structurally complex, characterized by unique functionality and marked biological activities, but the biotechnological potential of this precious resource is still largely underexplored. This knowledge gap stimulates the scientific community to further continue and deepen research in the field of bioprospecting, also through the development of new extractive methods and/or technological processes [23,24], in addition to those currently available. Valorization routes for such raw material can provide a great additional income chance for companies active in the production of fine chemicals, such as pharmaceuticals, allowing to give a commercial value to a biomass otherwise lost. To this aim, technological solutions with moderate operating costs could provide a route for a valuable processing system [23,24,25].

Compared to other reviews available on this subject, our review gives emphasis to novelty elements for the valorization of biomasses, such as the potential of gut microbiota as a source of probiotic candidates, and the use of fishery discards for energy production in a biorefinery perspective, in the general context of circular economy.

## 2. Typology of Fishery By-Catch and Processing By-Products

Fishery by-catch and processing by-products include different typologies of tissues and organs, including head, tails, skin, scales, bones, viscera that account for variable percentages of total fish body (Table 2).

Solid materials of fishery post-processing include head, tails, skin, gut, fins and frames. About 30–50% of the meat is usually left during whole fish filleting; the remaining, about 4–5% of skin, 21–25% of head, and 24–34% of bones account for more than 45% of the whole fish body and are largely unutilized [17].

After chemical, enzymatic, and/or microbial treatment, different bioactive compounds can be extracted from fish heads, skins and bones [23]. In order to evaluate potential applications of the heads of skipjack tuna (*Katsuwonus pelamis*), their proximate composition and amino acids, fatty acids, and carnosine contents were analysed [26]. Albeit they contain low proteins and lipids (18.47 g/100 g FW protein and 4.83 g/100 g FW lipid), essential amino acids constitute more than 40% of total amino acids; skipjack tuna heads also contain 1761 mg/kg FW carnosine, suggesting that they are a source of high-quality proteins.

Fishery by-catch and by-products contain huge quantities of scales, representing on average 2% of fish body weight [25] and their management as wastes is difficult, since fish scales are poorly biodegradable. Fish scales have a surface layer containing hydroxyapatite [Ca_10_(PO_4_)_6_(OH)_2_], calcium carbonate and an inmost layer made up of type I collagen, as well as of low concentrations of Ca, Mg, P, Na, S. Harikrishna et al. [27] studied their use as a substrate for the production of alkaline protease by *Bacillus altitudinis* GVC11 and of an hydrolysate rich in amino acids.

Fish bone is obtained after removal of muscle fillet from the frames; a superheated steam treatment allows a better bone recovery and reduced loss of soluble components from fish tissue [14]. Fish bone provides a valuable source of organic components (collagen and gelatin); inorganic substances contained in fish bone are mostly represented by calcium phosphate and hydroxyapatite. Therefore, fish bone is considered as a potential source of calcium, which is an essential element for human health. However, its incorporation as a food ingredient requires a preliminary conversion into an edible form through thermal treatment with water and acetic acid. Attempts have been made to use hydroxyapatite isolated from fish bone in substitution of synthetic hydroxyapatite in a range of medical and dental applications (for rapid bone repair after traumatic events [28]) due to their similar chemical composition. Fish hydroxyapatite shows a high mechanical resistance under physiological conditions, and is thermodynamically stable at physiological pH. It is isolated from fish bone by thermal treatment at a very high temperature (1300 °C), so as to increase the mechanical resistance of its structure and provide a biocompatible material.

Fish skin is considered as a source of collagen and antimicrobial compounds that contribute to provide a first barrier against pathogens’ attack [29]. Mucus covering the skin is secreted by goblet, sacciform, and club cells and contains several antimicrobial factors, such as proteins, lysozyme, immunoglobulin and lectins [30].

Fish viscera, including stomach, gut, liver, account for up to 12–18% of the whole fish total body; as for their composition, proteins represent 15% of the total weight of viscera. They are recognized to be an important source of enzymes and also of probiotic candidates that can be isolated from fish guts, as extensively reported below [19].

## 3. High Value Compounds (Active Metabolites or Bioactive Products) from Fishery By-Catch and/or Processing By-Products

The composition of fish bodies is largely variable, depending on several factors (species, age, nutritional status, sex, season and health). However, a large fraction is represented by 15–30% protein (higher in salmon and lower in flounder), 0–25% fat (higher content in mackerel and lower in cod) and 50–80% moisture as well as ash [17]. The proximate composition of fish body is shown in Table 3.

Marine by-products from the fish processing industry and fishery by-catch are an important source of bioactive compounds, such as proteins (58%), proteins, amino acids and peptides, and several enzymes, collagen, gelatin, fat (22%), among which polyunsaturated fatty acids, monosaturated acids, palmitic acid and oleic acid, ash, chitin, vitamins and others compounds are of great interest for their high market value [13].

The content and mean market value of high value components obtained from fishery by-products is reported in Table 4.

### 3.1. Fish Proteins

Fish frames contain significant amounts of muscle proteins; easy digestible and with high nutritional properties, due to the presence of essential amino acids like lysine, valine and phenylalanine [16]. Fish proteins derived have a well-balanced amino acid composition, compared to other animal protein sources. Enzymatic hydrolysis is used to extract proteins and amino acids from fishery discards as an option to their disposal [31].

Fish protein composition varies depending on the fish species and season. Fish protein is generally utilized as fishmeal, fish sauce and silage [17].

Fishmeal from pelagic fish is the most widely used product obtained from fishery by-catch, and has an average market value of 46 Euro/tons [25]. It has excellent nutritional properties, with a high content of digestible protein, balanced essential amino acids, fatty acids, minerals, and vitamins; its main properties (amino acid profile, digestibility, and palatability) vary in relation to the raw material and production process employed [25]. Fishmeal is mostly used as the main source of protein in aquaculture feeds [32].

Fish sauces, widely consumed all over the world, are obtained by salt fermentation of pelagic fish or fishery by-products [17]; they are used as an important animal protein supplement in human diets.

Fish silage is a liquid product resulting from the liquefaction of the whole fish body or of a part; it represents a protein source with excellent nutritional properties for animal feeding [17].

### 3.2. Bioactive Peptides

Proteins extracted from the fish muscle contain several peptides with antihypertensive, antithrombotic, immune modulatory and antioxidative properties [33].

Proteins from the enzymatic hydrolysis of the fish muscle (protein hydrolysate) contain many biologically active peptides with precious nutritional and functional properties [17]. The bioactive peptides obtained from the fish muscle show also anticoagulant and antiplatelet properties [34].

Caruso et al. [35] found that the mucus secretions, biological fluids and kidney of sea bass (*Dicentrarchus labrax*, Moronidae), European eel (*Anguilla anguilla*, Anguillidae) and blackspot seabream (*Pagellus bogaraveo*, Sparidae) showed antibacterial, agglutinating and hemolytic activities, suggesting they are an interesting source of bioactive compounds. Antibacterial activity against *Photobacterium damselae* subsp. *piscicida* was detected in the kidneys of sea bass. Hemolytic properties against sheep red blood cells were observed in the mucus of sea bass and blackspot seabream. The sera of sea bass and eel showed also agglutinating activity against *Pseudomonas aeruginosa* and *Staphylococcus aureus*; while the mucus of sea bass was able to agglutinate isolates of *Vibrio fluvialis*, *V. alginolyticus* and *A. hydrophila*.

### 3.3. Fish Protein Hydrolysate (FPH) 

Fish protein hydrolysate (FPH) is a concentrated and purified form of proteins developed by cleavage of molecular bonds through biological processes; the enzymatic hydrolysis of protein can yield a by-product rich in nutrients and bioactive peptides [20,36]. FPHs have attracted much attention as compounds with pharmaceutical and industrial application due to their antioxidant, antihypertensive, immunomodulatory, antimicrobial, neuroactive properties, and mineral or hormone regulating abilities and with a good balance of essential amino acids, though there is little to no prospect for human use as a food or as an additive in the food industry. The enzymes used in the food industry for the preparation of FPHs are mostly carbohydrates, proteases and lipases. Alcalase, an endoprotease extracted from *Bacillus* spp., is widely used for the production of a FPH with high solubility and digestibility.

The technology of FPH production from fishery by-catch is considered as a cost-effective way to valorize these underutilized resources. The final product of this process should have a low lipid content, therefore many studies deal with the de-fattening of the raw material before the hydrolysis process, which potentially could improve the production process.

Monkfish by-products (heads and viscera) derived by on-board processing were used to produce FPHs [37]. The optimal proteolytic digestion was found at a temperature of 57.4 °C, pH 8.31, for 3 h in presence of 0.05% of alcalase. Under these conditions, the yield of digestion was higher than 90% for heads and viscera; the soluble protein content was lower than 45 g/L, the percentage of essential amino acids was 40–42%. Antioxidant properties were observed in the viscera FPH, while antihypertensive ability was higher in head FPHs.

Navarro-Peraza et al. [38] have recently reviewed the biological and structural properties of FPHs from both solid materials (i.e. skin, head, viscera, trimmings, and bones) and wastewater derived from operations such as washing, thawing, cooking and fishmeal production, in the perspective of their valorization as hydrolysates and bioactive peptides.

The extraction of FPHs from *Leiognathus splendens*, a bycatch fish with low commercial value, was optimized by Prabha et al. [39]: optimum conditions were obtained at a temperature of 50 °C for 90 min and a pH 5, resulting in the highest degree of hydrolysis (37.9%) and high protein (74%), and low lipid (1.37%). This FPH contains low molecular protein peptide (20 kDa) and high levels of essential amino acids, and its composition fulfills the nutritional requirements for poultry and human adults.

Ishak and Sarbon [40] suggested that peptides with low molecular weight and short amino acid sequences act as potent bioactive peptides. These authors referred to the production of purified bioactive peptides from various FPHs via enzymatic hydrolysis, followed by the purification of bioactive peptides by ultra- and gel filtration, as well as by a RP-HPLC stage after enzyme screening and optimization. 

Vázquez et al. [41] evaluated the suitability of various by-products (heads, trimmings, and frames) obtained from rainbow trout and salmon processing as substrates for the production of FPHs, as potential protein ingredients of aquaculture feeds. Initially, enzymatic conditions of hydrolysis were optimized; for the hydrolysis of heads optimal conditions were 0.1% (*v/w*) of alcalase, pH 8.27, at 56.2 °C for 3 h and agitation of 200 rpm for rainbow trout and 0.2% (*v/w*) of alcalase, pH 8.98, 64.2 °C for 3 h and agitation of 200 rpm for salmon. The protein quality of FPHs was excellent in terms of digestion of wastes (Vdig > 84%), high degrees of hydrolysis (Hm > 30%), high concentration of soluble protein (Prs > 48 g/L), good balance of amino acids, and almost full in vitro digestibility (Dig > 93%). The FPHs obtained from trimmings + frames (TF) of salmon showed the highest protein content compared to that from other species. Hydrolysates of rainbow trout heads and salmon TF led to the highest percentages of small peptides (0–500 Da). Besides FPHs and bioactive compounds (antioxidant and antihypertensive), fish oils were also recovered from salmonid wastes.

Mazorra-Manzano et al. [42] reported the production of a FPH from Pacific whiting (*Merluccius productus*) muscle through autolysis of a minced homogenate (8% protein) at pH 7.0 and 60 °C, conditions providing the highest endogenous proteolytic activity. FPH showed better emulsifying properties than sodium caseinate (SCA) at pH 4.0 (*p* ≤ 0.05) or bovine serum albumin (BSA), suggesting it is possible to reach similar or better functional properties than those of functional ingredients, such as SCA and BSA.

During tuna processing, the black muscle of *Sarda orientalis* was used to produce a FPH at a temperature of 56 °C for 4 h [43]: the FPH obtained at the optimal conditions resulted in an average degree of hydrolysis of 7.72% in response to the required range of 5–20% and could be used as a nutritional source of nitrogen and as a potential antioxidant compound.

Villamin et al. [44] have recently reviewed the potential of fishery discards, especially viscera, as a source of native proteins and hydrolysates, outlining their production process, chemical composition and functional and bioactive properties that are important to the agricultural, cosmetic, pharmaceutical, food and nutraceutical industry.

The production, functional and bioactive properties and potential applications of FPHs from fish viscera have been detailed by Villamil et al. [44].

### 3.4. Antimicrobial Peptides Isolated from Fish

The presence of conventional and unconventional antimicrobials (AMP) in fish has also been reported [45,46]. The first family of peptides with these properties discovered in fish was that of α-helical pardaxins, isolated from the skin glands of Red Sea Moses sole, *Pardachirus marmoratus*, active against Gram-positive and Gram-negative bacteria with pore-forming activities. Most fish α-helical peptides are members of the piscidin family, which includes the pleurocidins and piscidins. The first are 25-residue peptides first isolated from the skin mucus of winter flounder, *Pleuronectes americanus*, with a broad-spectrum of antimicrobial activities, able to inhibit DNA, RNA and protein syntheses. The second ones are 22-residue peptides first purified from skin and gills of hybrid striped bass (*M. saxatilis* x *M. chrysops*) and also found in other Perciformes. Belonging to the piscidin family are dicentracin from the European bass, *Dicentrarchus labrax*, chrysophsins from red sea bream, *Chrysophrys major* andepinecidin from the orange-spotted grouper, *Epinephelus coioides*. All piscidins show broad-spectrum antimicrobial activity, probably killing cells via toroidal-pore formation, but their topical application use is not approved due to their hemolytic and cytotoxic properties.

### 3.5. Enzymes

Fish viscera are among the most important fishery by-products, due to their content of digestive enzymes, many of which exhibit high catalytic activities at relatively low concentrations, and high stability in a wide range of pH. Consequently, these enzymes have a wide range of potential industrial applications [13,25,47], including seafood processing for collagen removal. 

The most important proteases in fish viscera are pepsin (an aspartic protease) and serine proteases (trypsin, chymotrypsin, collagenase, and elastase) [17,20,47,48,49,50,51,52]. Enzymes can be endogenous (pepsin, trypsin, chymotrypsin, collagenase and elastase) or produced by the fish microbiota; they represent a large percentage of bioactive compounds present in fishery by-catch or by-products and are commercially extracted on a large scale [17], but to date their potential application has been only partially disclosed. Fish proteases are the most used enzymes, probably in relation to the wide range of applications of these proteins, mainly alkaline proteases, in food, detergents, pharmaceuticals industries [50,53,54,55,56,57,58].

Among digestive proteinases detected in the pyloric caeca and intestine of fish, trypsin (EC 3.4.21.4) is a serine proteinase, able to catalyze the hydrolysis of peptide bonds at the carboxyl group of arginine and lysine residues and to activate all pancreatic enzymes [50,51,56]. The use of trypsin extracted from fish viscera has recently increased significantly, due to its high stability and activity at high temperature and pH.

Trypsin and trypsin-like enzymes have been isolated from several fish species both living in cold habitats such as chinook salmon (*Oncorhynchus tshawytscha*) [59], and warm water fish such as Monterey sardine (*Sardinops sagax caerulea*) [52], true sardine (*Sardinops melanostictus*) [60], sardine (*Sardina pilchardus*) [61], striped seabream (*Lithognathus mormyrus*) [62], bogue (*Boops boops*) [63], sardinelle (*Sardinella aurita*) [64] and yellowfin tuna (*Thunnus albacores*) [65]. The recovery of proteolytic enzymes from fisheries by-products could, therefore, contribute to produce novel low-cost proteinases for industrial application and simultaneously to solve the issues of fishery discards management [66].

The extraction and purification of proteolytic enzymes from fish viscera is generally carried out by various separation techniques such as salt and organic precipitation, chromatography, or phase separation by an aqueous two-phase system. However, most of these methods are time consuming, too expensive, and require technical high technical skills [66]. Proteases were isolated by Murthy et al. [67] from little tuna (*Euthynnus affinis*) of different habitats using acetone, ethanol and ammonium sulfate fractional precipitation and characterized. The proteases showed higher specific activity at 40% saturation for ammonium sulfate fractional precipitation and their specific activities were 18.19 U/mg. The pancreas of this species (*E. affinis*) is a source of lipase; Mardina et al. [68] extracted this molecule, which showed an enzymatic activity of 11.962 U/ml with optimum pH 9.

Chitinases as well as chitosanases can also be isolated from digestive tract and other organs of some marine fish species [69]. These enzymes promote the recovery of chitin and chitosan from marine byproducts necessary for a wide array of biomedical applications.

### 3.6. Collagen and Gelatin

Collagen is the major structural protein of fish skin and bones, representing 30% of the total protein content [20,25]. Marine collagen is a promising biomaterial for biomedical applications [70,71]. Collagen is the generic name for a superfamily of proteins and accounts for around 30% of the whole protein content in most vertebrates. It is the main component of the extracellular matrix (ECM) in connective tissues (skins, tendons, ligaments and bones). It is used in many health-related fields and in food processing. Collagen is classified according to its structural features; the two only classes present in fish (I and II), are proper for cosmetic and regenerative medicine (wound healing) [72].

Fish skin, tendons, cartilage, bone and connective tissue contain both collagen and gelatin which can be extracted and used in food and pharmaceutical products. Fish gelatin is also known to possess antioxidant properties related to its unique sequence in terms of glycine–proline–alanine components; it possesses characteristics similar to porcine gelatin, and may also be considered as an alternative to mammalian gelatin for use in food products [13,72,73]. An important advantage of using fish derivates relies on the fact that both these products are, compared to the bovine derivates, free of risks for the transmission of spongiform encephalopathy. Collagen and gelatin are two different forms of same macromolecule; being gelatin a partially hydrolyzed form of collagen in a denaturated state. Gelatin is a fibrous protein obtained from the heat denaturation of collagen. Fishery discards contain collagen at a high extent (around 30% wt) in skin, fins and bone. The limiting factor for collagen industrial demand, round 320,000 tons/year, is the high cost.

Lim et al. [71] reviewed the species used as a source for the extraction of collagen from fishery by-products, such as skins and bones of Spanish mackerel (*Scomberomorus niphonius*), swim bladders of yellowfin tuna (*Thunnus albacares*), scales of seabass (*Lates calcarifer*), swim bladders and scales of miiuy croaker (*Miichthys miiuy*). The structural and thermal stability of marine derived collagens was found to be weaker than those of mammal, due to their lower proline and hydroxyproline contents, however, they are more easily hydrolyzed by proteases compared to mammal collagens and are suitable to be further processed to produce bioactive peptides. Therefore, bioactive peptides prepared using marine derived collagens and gelatins have attracted broad attention due to their various promising applications.

Collagen peptides from the hydrolysate fraction (F7) of Spanish mackerel (*Scomberomorous niphonius*) skins have been isolated and their anti-oxidant activity in vitro has been shown [74].

### 3.7. Chitin and Chitosan

Chitin is a structural component of fish scales, other than shrimp and crab shells and squid pens [25]. Marine chitins have been utilized for the production of vast array of bioactive products, including chitooligomers, chitinase, chitosanase, antioxidants, and antidiabetic compounds and prodigiosin, a potential candidate for cancer drugs [75,76,77].

Chitosan is commercially obtained mainly from chitin by the deacetylation process performed by the addition of alkali solutions. Chitin and chitosan are ubiquitous marine polysaccharides; over the years, they have attracted a great deal of attention in food, pharmaceutical and health applications due to their distinctive biological and physicochemical characteristics [25]. The adhesive nature of chitin and chitosan, together with their antioxidant and antimicrobial properties, is a very important property for biomedical and pharmacological applications and in food industry in food additives and packaging materials [78].

Environmentally friendly processes that combine various microbial, chemical, enzymatic and membranes strategies and technologies to extract and purify from marine by-products chitin/chitosan, as well as chondroitin sulfate, hyaluronic acid were reviewed [79].

### 3.8. Lipids

Almost 50% of the body weight generated as by-products during the fish processing is a great potential source for good quality fish oil, which can be used for human consumption or production of biodiesel [17,79]. Indeed, fishery by-products contain lipids (2–30%), in the form of fish oil, whose concentration varies depending on the fish species. 

The fish oil contains two main polyunsaturated fatty acids, eicosapentaenoic acid (EPA) and docosahexaenoic acid (DHA), that are classified as omega-3 fatty acids. They are mainly found in the marine animals which have high polyunsaturated fatty acid content [17]. Omega-3 fatty acid concentrates are of great interest for the pharmaceutical and food industries, for the production of drugs with enhanced performance and nutritional supplements [25].

Rubio-Rodriguez et al. [80] compared supercritical fluid extraction using carbon dioxide with other conventional fish oil extraction processes, such as cold or enzymatic extraction, and wet reduction, showing this method is effective in reducing fish oil oxidation, especially when fish oil is rich in omega-3, such as salmon oil. Viscera from farmed gilthead sea bream (*Sparus aurata*) and sea bass (*Dicentrarchus labrax*) and also oil extracted from these by-products have been studied to assess their suitability as DHA sources by chromatography, obtaining a highly purified DHA fraction (>99.0% DHA on total fatty acids) [81].

Rincon-Cervera et al. [82] analyzed the fatty acid profiles of dried and raw by-products from Chilean farmed salmon and wild red cusk-eel and yellowtail kingfish, pointing out that Soxhlet procedure with n-hexane is effective to extract lipids containing EPA and DHA from dried by-products for nutritional or nutraceutical purposes. In addition, fish oil contained vitamins and essential minerals such as calcium, phosphorus, magnesium and trace elements.

### 3.9. Minerals

Fish bones contain 60–70% of minerals, including calcium, phosphorous and hydroxyapatite [13]. Generally, calcium is deficient in most of the regular diets and to improve calcium intake, consumption of small whole fish can be nutritionally valuable. The fish bones obtained from fish processing operations can be used to provide calcium. In order for bones to be a fortified food, they should be converted into edible form by softening their structure with thermal treatment with water and acetic acid solutions or by superheated steam cooking [83].

Fish bones are a very good source of hydroxyapatite which can be used as a bone graft material in medical and dental applications. The important properties of hydroxyapatite are related to its stability thermodynamic stability at physiological pH [84].

## 4. Fishery By-Catch or Processing By-Products as a Source of Probiotics

The skin, gills and gastrointestinal tract (GIT) are the major pathways for microbial entry in fish and represent the sources of bacteria which can play a probiotic role for the host health [85].

The term probiotics which means “for life” from the Latin word ‘*pro*’ (for) and the Greek word ‘*bios*’ (life) was introduced in the early 20th century by the Nobel Prize-winning Elie Metchnikoff, who hypothesized that the long healthy lives of Bulgarian peasants were the result of their consumption of fermented milk [86]. The Joint Food and Agriculture Organization of the United and World Health Organization (FAO/WHO) have stated that probiotics are “live microorganisms which, when consumed in adequate amounts, confer a health benefit on the host” [87] by providing benefits to the host primarily via the direct or indirect modulation of the intestinal microbiota. According to a wide scientific literature, a probiotic should be of autochthonous origin, have a great ability of competing, predominating with resident microbes, persisting in the enteric environment and be safe [88].

The beneficial effects of probiotics on growth, feed conversion, enzyme activity, immune response, stress, disease resistance and their use has a long tradition in animal husbandry [89]; however, the probiotic concept has been successively transferred to the fish farming industry; many works [86,90,91,92,93] describe the functions of fish gastrointestinal microbiota, the potential application of probiotics in finfish aquaculture, as they exert a beneficial effect via a wide array of actions, such as competition for adhesion sites, essential nutrients and production of antagonistic compounds against pathogens. Probiotics can be introduced in commercial aquaculture as live microbial cultures or in the form of bacteria biofilm or by incorporating them into formulated fish diets to achieve a high colonization in the fish GIT [88,92,94,95,96]. Marine probiotics are symbiotic or associated microbes and most of them originated from algae, sponge, invertebrates and fish organs [97].

### 4.1. Fish Organs Probiotics 

The earliest studies of the microbial communities associated with fish date back to the late 1920s. Since these pioneering investigations, much research effort has been dedicated to describing the microbial communities present in Teleost fish [85]. The beneficial effect of marine bacteria has been ascribed to the production of several molecules which constitute part of cellular membrane, cell wall or are products of the microbial secondary metabolism. Table 5 presents a wide range of bacterial species, isolated from fish gut, gills and skin and gives an overview on different studies focused on the micro-symbionts from the major economically important marine host fish species, their probiotic activity and, for some entries, it highlights the specific biomolecule/type that can be considered an added-value for a biotechnological potential. References are reported as well for the description of methods for the identification of probiotic activity (using cultured bacterial cells or cell free culture surnatants or crude extracts) and for the chemically characterization of some bioactive molecules, which have been extracted/isolated, purified. 

As shown in Table 5, a large number of microbial species have been isolated and evaluated as probiotics: Gram-negative (*Enterobacter* sp.; *Aeromonas* spp.; *Plesiomonas shigelloides*; *Hafnia alvei*; *Citrobacter freundii*; *Shewanella xiamenensis*; *Vibrio* spp.; *Photobacterium* sp.; *Agrobacterium* spp.; *Azospirillum orizae*; *Pseudomonas* spp.; *Psychrobacter* sp.; *Acinetobacter* spp.; *Sphingomonas* spp.; *Erwinia persicina*; Cyanobacteria), Gram-positive (*Carnobacterium* spp.; *Enterococcus faecium*; *Lactobacillus pentosus*; *L. fructivorans*; *L. delbrueckii*; *Bacillus* spp.; *Brochothrix* sp.; *Leuconostoc* sp.; *Micrococcus* sp.; *Macrococcus* sp.; *Microbacterium;* spp., *Paenibacillus* spp., *Arthrobacter arilaitensis*; *Mycobacterium* sp.; *Anoxybacillus* spp.; *Staphylococcus* sp. *Actinobacteria*), Fungi (*Aspergillus spp*.; *Candida santamariae*; *Trichosporon laibachii*) and Yeasts (*Debaryomyces hansenii*, *Pichia* spp., *Kodamea ohmeri*; *Candida* spp.; *Saccaromyces cerevisiae*; *Leucosporidium* spp.; *Rhodotorula* spp.).

### 4.2. Fish Gastrointestinal Tract (GIT)

#### 4.2.1. Bacterial Activities

A complex and variable bacterial community inhabits the gastrointestinal tract of fish, playing a role for the host’s health and quality and takes part in various digestive processes thanks to the synthesis of bioactive compounds [98,99]. The most studied activity is antimicrobial, which has been detected in the intestinal microflora of different marine fish species as *Lates calcarifer*, *Epinephelus coioides*, *Mugil cephalus*, *Onchorincus mykiss*, *Sparus aurata* [91,98,100,101,102,103], but also an immunomodulatory effect and an activity on growth performance were evidenced in the gut of *Sparus aurata*, *Dicentrarchus labrax*, *Solea solea* [88,94]; Fidopiastis et al. [104] found that zebra perch *Hermosilla azurea*, an herbivorous marine fish, produced in all gut regions, microbial digestive enzymes, like polysaccharides and different short chain fatty acids (SCFAs) as acetate, propionate, valerate, butyrate, isobutyrate, which represent an important source of energy for herbivorous [105,106] and are known (butyrate) for antibacterial and antitumoral activities [107]. The pioneering studies of [105] on the production of eicosapentaenoic acid (20:5 (n-3)) EPA by *Vibrio pelagius* have been later confirmed in recent studies pointing to these compounds as cardio-protective agents and experimented for neuroprotection activity [108]. Furthermore, other studies described the amylolytic, cellulolytic, chitinolytic [109,110,111], proteolytic and lipolytic enzyme activities in different region of the digestive tract of *Mugil cephalus*, *Anguilla japonica*, *Salmo salar* [112,113], *Gadus morhua* [114], *Lates calcarifer* and *Chanos chanos* [115] and highlighted their role to improve the nutritional value of feeds [116,117].

Recent researches show the intestinal microflora of gilthead seabream is a novel source of bioactive compounds from *Pseudomonas* spp., *Aeromonas* spp., *Acinetobacter* sp., *Sphingomonas* spp. strains. These secondary-metabolism products called biosurfactants (BSs) showed to exert both surface and antimicrobial activity against marine pathogens *P. damselae subsp. damselae*, *V. anguillarum*, *S. aureus* and *A. hydrophila* and were chemically characterized as glycolipid/rhamnolipid [98]. 

Microbial surfactants have a large range of activities, from antimicrobial, antifouling, anti-adhesive, to antithrombotic, anti-cancer and represent new multifunctional drugs of the 21st century which are attracting a great interest due to their biocompatibility, versatility and applications in pharmaceutical, nutraceutical, cosmeceutical, industrial and environmental field [118,119,120]. Floris et al. [121] presented an updated overview of several BS types from marine microorganisms, the source of isolation, methods and screening procedure for their production and test. However, marine BS have not been widely explored yet, mainly due to the issues associated with the isolation and growth of producing microorganisms. The use of culture-independent techniques (metagenomics) constitutes a promising approach to study the genetic resources of marine microorganisms, aimed at discovering novel high-value biologically active molecules from marine matrices like marine discards [120]. In this respect, genetic engineering could be a boon for improving in the yield of bioactive metabolites as the biosynthetic pathways can be manipulated through recombinant DNA technology. In addition, researchers are now trying for heterologous expression of biosynthetic gene clusters in other organisms, which would not only increase the production levels but also speed up the growing process of the manipulated organisms [97,122].

#### 4.2.2. Yeast Activities

As regards the yeasts typically identified from the fish GIT, they belong to the Ascomycota (*Debaryomyces* sp., *Candida* spp., *Pichia*, *Saccaromyces* sp.), or Basidiomycota (*Leucosporidium* sp.; *Rhodotorula* spp.) (see Table 5). According to [123], the natural occurrence of yeasts in the mucus may generally be considered as commensalism in fish gut, in spite of the reported cases of pathological infections of immune compromised fish individuals, which are mainly due to opportunistic strains. Yeasts represent a rich source of bioactive substances like β-glucans, mannan oligosaccharides (MOS), which are well-documented immunostimulants and prebiotics, respectively [85]. Different experiments of intestinal colonization by yeasts were carried out in some marine fish aquaculture and their competition with other intestinal microorganisms was highlighted [123]. Gatesoupe et al. [116] stated that the yeast produced extracellular proteases, siderophores and bound lactoferrin; iron availability is the key for fish microbiota activity and it would be worth investigating their potential against several fish diseases. The effects of live yeast on modulation of fish immune response, metabolism and growth have been experimented in gilthead sea bream *Sparus aurata* [124] and European sea bass *Dicentrarchus labrax* larvae for which *D. hansenii* HF1 improved survival and vertebral conformation, possible due to the observed maturation of the digestive system [125].

### 4.3. Fish Skin and Gills

Research on the microbiota associated with the skin of fish dates back to the 1920s. This early research was conducted on skin epidermal samples and “slime” (i.e., mucus) to determine the role of microbes in fish spoilage [126]. The mucous layer of the epithelium provides the epidermal immunoglobulins, defensins, lysozyme, lectin-like agglutinins and a variety of antimicrobial peptides that provides a broad spectrum of antibacterial activities [85]. However, accurate determination of the microbes on the skin of fish is difficult because of contamination of the epidermal tissues during the capture and handling of the fish. Several groups of microorganisms are typically reported to be components of the skin microbiota [127]. 

Table 5 shows the most common microbial species, although most of the available information about fish skin deals with the bacterial components as for fungi and yeasts. As for the microbiota associated with fish gills, there still lacks a comprehensive knowledge, since this organ is a difficult habitat for microbes due to the continuous water flux passing over. Nevertheless, the presence of bacteria with antibacterial properties on the mucosal surface of the gills has been reported [128]. Gram and Ringø [127] gave a prospect of fish gills microflora and describe their probiotic effect on fish and larval survival. The most frequent bioactivity is antibacterial or antagonism and competition for adhesion sites and/or nutrients [129] while a low presence of yeasts is reported in the skin/gills of fish [85]. The microbial communities on the gills are as abundant or even more than those present on the skin, and as abundant or less than those reported in the GI tract and many factors as diet, environmental factors, mucous layer of the epithelium etc., have been reported to affect their composition or abundance [85,130].

## 5. Methodologies/Technologies of Extraction

Ideia et al. [23] recently reviewed the methods used for the extraction and characterization of fish processing industrial routes, as well as the potential applications of the added value products extracted by fish by-catch or processing by-products, focusing on high added value compounds such as hydroxyapatite, collagen, gelatin, lipids, enzymes, hydrolysates and bioactive peptides. New technologies for the use of animal food processing wastes have also been reviewed by Shen et al. [132]. To date, however, exploitation of the potential value of fishery discards is far from being resource-efficient and cost effective, due to inefficient or expensive extraction strategies. Moreover, different processing conditions, such as source material, composition, matrix properties, extraction solvent type, solvent concentration, process temperature, pH conditions, pressure and overall extraction period, all can affect the properties and the yield of extraction of bio-active compounds/biomolecules and the process efficiency, therefore implementation and standardization of extraction procedures is urgently required.

The use of innovative green technologies such as ultrasound-assisted extraction and supercritical fluid extraction (SFC) has recently been suggested [24] as an interesting alternative to the methods conventionally used for the valorization of seafoods and their by-products, preserving and even enhancing the quality and the extraction efficiency, as well as minimizing functional properties’ losses of the bioactive compounds extracted from marine by-products. The SCF extraction has been largely applied for commercial products in food industry, however, a wide panel of operative conditions allows to tune it for more specialized applications in the nutraceutical and pharmaceutical applications, for high purity products of high added value products [133]. In the biorefinery scenario, SCF plays a central role, since it does not alter the biomass in the perspective of further extraction: for instance, in a process including the fish oil extraction followed by collagen or hyaluronic acid extraction from the residual solid from the fish oil extraction, the application of traditional methods for fish oil extraction, at high temperature with the use of organic solvents, would impair the quality of residual solid so that of the final products (collagen, hyaluronic acid). On the other hand, the high market value of final products (fish oil, collagen and hyaluronic acid) obtained with the scheme proposed for fishery discards processing by Serangeli et al. [134] justifies the use of expensive techniques that can in turn guarantee the final products quality.

Regarding the extraction of lipids (ω3 fatty acids), the source of ω3 long-chain polyunsaturated fatty acids (eicosapentaenoic acid (EPA) and docosahexaenoic acid (DHA) [135] is the fish oil, whose global production is around 800 to 900,000 tons/year, while the European production is round 190,000 tons/year [136]. Of this round 73% goes to aquaculture, 21% for direct human consumption and 6% for other purposes. The overall trend over the last 5–10 years was a fall in production while its demand is continuously increasing. It is therefore necessary to find alternative sources and also to explore new technologies to have a higher quality extraction, lower energy utilisation and pollutant emissions.

The use of discards rather than of virgin raw material represent a good trade-off between growing market demands and fish stocks maintenance. In addition, innovative extraction methodologies, such as supercritical fluid CO_2_ (SCF-CO_2_) extraction of fish oil and ω3 from fishery discards, would solve the impact of extraction methods with traditional solvents (such as n-hexane) [137]. Indeed, as a co-solvent of nonpolar lipids in SCF is generally used ethanol, which is however much more food compatible than hexane, a widely used solvent for extraction of the lipidic fraction from fish processing by-products.

CO_2_ is the ultimate green solvent since it shows low toxicity and cost, and it is not flammable. Its mild critical conditions (T_c_ = 304.15 K, P_c_ = 7.38 MPa) make it suitable to process thermo-labile components, such as polyunsaturated fatty acids. It is used to fish oil extraction and further fractionation, to produce ω3 concentrates enriched in EPA+DHA. Its advantages are: the low processing temperature reduces the effect of oxidation of polyunsaturated fatty acids; residual solvent contaminants (heavy metals and PCB) are strongly reduced compared to traditional methods (such as solvent extraction) [138].

The only drawback of the method is that the fish oil and ω3 concentrate formulations retain volatile compounds, which are responsible for bad odours. Encapsulation methods reduce this effect, along with lipids oxidation, improving the shelf-life of products along with their palatability [139].

With respect to the extraction of collagen, traditional processes to extract collagen from fishery discards require acidification and a salting-out step to solubilize the acid-soluble fraction; the extraction from bones require an additional pre-treatment of decalcification by means of EDTA [13].

SCF-CO_2_ is a technology for the extraction of compounds, being generally regarded as green solvents, being recently applied to the extraction of collagen from marine resources based on the pressurization of water with carbon dioxide, resulting in an acidic, hot and pressurized extraction environment. Among several advantages, it is a clean method that avoids the use of any organic solvents and reduces very significantly the amount of water required. Moreover, it can be performed in one single extraction step allowing the reduction in the extraction time and therefore in processing costs. It has been described as a successful technique to obtain purified collagen to be use in high-demanding industrial sectors, namely in biomedical context [140].

Innovative methodologies allow for the simultaneous extraction of lipids and proteins by means of food-grade switchable solvents, a very promising technique that would revolutionize the multiple extraction of bioactive compounds from residual biomasses [141].

Switchable hydrophilicity solvents (SHS) are a new class of solvents that are able to change their nature, from hydrophobic to hydrophilic and vice-versa by simple means, such as adding and removing CO_2_ at a low pressure from it. Usually these solvents are used in their hydrophobic form to extract hydrophobic solutes. Recently, the possibility to use both phases of these solvents has been explored to extract both hydrophobic and hydrophilic bioactive compounds from biomass [142].

## 6. Fields of Application of Fishery By-Catch or Processing By-Products

Depending on their unique structural and functional characteristics, marine-derived bio-active compounds/biomolecules can be exploited in different pharmaceutical (biomedical, nutraceutical), cosmetical, and biotechnological (chemical or industrial) application fields.

### 6.1. In Food and Nutraceutical Industry

Fishery discards provide an interesting source of high added value compounds, such as hydroxyapatite, collagen, gelatin, lipids, enzymes, hydrolysates and bioactive peptides, with great potential for different applications [23].

Fishmeal and fish oil represent the most valuable products obtained from marine by-products that are not used for human consumption [25]; it has been estimated that each year about 60% of fishmeal and 80% of fish oil of the world total production are consumed as aquaculture feeds and this percentage is expected to increase to warrant the future aquaculture growth [143].

Amino acids such as lysine, threonine, methionine and tryptophan are used in the animal feed industry to improve the nutritional quality of animal feeds; use in the food flavoring industry of amino acids from fishery by-products has also been suggested, such as glutamate, alanine, aspartate and arginine. 

Fishery discards have been considered as important sources of high value nutraceuticals and other ingredients such as natural food additives, bioactive compounds, nutraceuticals [31]. Since fish feeding require supplementation of vitamins, minerals, and antioxidants [5], this could be provided by fishery by-products.

Marine bioactives appear to fit the criteria established for functional food ingredients, since they are naturally occurring compounds widely available, and their isolation/extraction is relatively cost-effective.

### 6.2. Applications in Pharmaceutical and Biomedical Industry

Evident effects for human health are related to the use of fish skin and viscera as a source of antimicrobial molecules and probiotic strains, respectively.

Antioxidant activity has been reported for FPH prepared from several marine species such as tuna, mackerel, yellowfin sole, and Alaska pollock [144,145,146]. Peptides isolated from fish can be derived from muscles, skin, scales, bones, and other tissues. The antioxidant nature of FPHs is mainly dependent on peptide size and amino acid composition [147].

Giannetto et al. [148] have recently shown the high nutritional properties and anti-inflammatory and anti-oxidant activities of protein hydrolysates obtained from anchovy *Engraulis encrasicolus* (APH) highlighting the use of bioactive molecules from fishery by-products and their use as potential nutraceuticals in food and pharmaceutical industries.

Fish oil, in relation to its content of EPA and DHA, is beneficial for human health. Fish consumption has been reported to be active against obesity; indeed, DHA and EPA prevent obesity by inhibiting key enzymes responsible for lipid synthesis. Evidence that omega 3 counteract obesity was reported [149]. Moreover, fish oils have been tested as alternatives for nonsteroidal anti-inflammatory drugs.

The omega-3 fatty acids prevent cardiovascular diseases, reducing blood pressure, and also protecting from arrhythmia. Populations that consume the most marine products like Alaskans and the Japanese suffer less from heart diseases. Additionally, Mediterranean people, who consume high amounts of seafood (as part of a Mediterranean diet) showed reduced heart diseases mortality because omega-3 fatty acids decrease the risk factors associated with triglyceride concentrations, blood pressure, platelet aggregation, and heart arrhythmias [150].

Seafood has been reported to have neuroprotective effects; fish consumption was associated with a reduced risk of ischemic heart disease and stroke mortality, protecting against neurodegenerative diseases [151,152,153,154]. A reduced intake of omega-3 fatty acids or fish consumption was correlated with an increased risk for age related cognitive decline or dementia diseases. Calon and Cole [155] reported the association between DHA consumption or high DHA blood levels and a lower risk of developing Alzheimer’s disease in elder people.

A neuroprotection potential has also been shown for marine glycosaminoglycans such as hyaluronic acid from tuna eyeballs and shark fins from codfish bones and chondroitin sulfate from fish skin cartilage, which offer therefore perspectives of application in medical fields [108].

Improved survival in cancer patients thanks to diets rich in fish products has also been observed. Several studies have been conducted to prove the relation between cancer prevention and fish consumption. Picot et al. [156] have studied the antiproliferative activity of FPH in vitro on 2 lines of human breast cancer cells. In mice bearing the Lewis lung carcinoma, a diet rich in omega-3 fatty acids fish oil suppressed tumor growth and reduced the metastatic biomass. Fish oil has been shown to increase the effectiveness of cancer chemotherapeutic drugs.

### 6.3. Applications in Fish Processing or Other Industrial Uses

Several high-value compounds, such as hydroxyapatite, collagen, gelatin, lipids, enzymes, hydrolysates and bioactive peptides, can be extracted from fishery discards, with great potential for different applications [23].

Enzymes extracted from fishery by-catch or by-products can be used for seafood processing and production of FPH [157] or can found an application in laundry process [53,61].

Fish by-products can also be converted into biodiesel [158]: biodiesel consists of monoalkyl esters of fish oils that can be synthetized from edible, non-edible and waste oils. It is a non-toxic, biodegradable and renewable energy source and can be produced chemically or enzymatically and chemically (using alkali such as NaOH as catalysts in the conversion of triacylglycerol (TAG) to methyl esters (biodiesel), with short reaction times (4–10 h) and disadvantages, such as high reaction temperature, soap formation, waste generation. Enzymatic trans-esterification is performed mostly by lipases derived from *Pseudomonas* spp. or *Candida* spp., but require long reaction times (12–24 h) and is expensive, conversely, it does not require high temperature and there is no soap formation.

Ahuja et al. [159] states that fishery discards can also be used for the production of dry and liquid fertilizers, fish compost and digestates, providing nutrient inputs to organic farming systems.

Aquaculture and fishery industry are important sectors of the economy of many Countries; their recent growth has resulted in the generation of large amounts of wastes, most of which are discarded, in spite of their active and functional properties, with a significant impact on the environment where they are discharged [160].

Currently, several relevant industries incorporate bioactive fish molecules (proteins, lipids to minerals) in numerous products. The low prices and high quality of the raw material determine the use of these biomolecules. The high demand for fish originates an inadequate overexploitation of marine resources. In most cases, the fish processing industry uses part of the fish while the rest is discarded. Fish stocks are finite and highly overfished globally, so it is necessary to valorize all biomass in a sustainable way. The amounts of under-utilized residues generated by fish processing industries can create serious environmental issues and have led researchers and industries to actively seek for alternative strategies to use fishery by-catch or by-products from fish transformation as raw materials for industrial processes.

The biorefinery concept in the search for sustainability is thriving with the use of all substrate to obtain products to be used by different industries whilst single product extraction ceases. Research has been focused on the use of innovative, economically and environmentally sustainable extraction methods to preserve the biological activity of the molecules and respond to the increasing awareness of consumers in product related issues. These pioneer methods can transform fishery discards into added-value by-products using an efficient and viable economic strategy. This review presents in this context the use of different by-products and techniques to extract highly desirable biologically active compounds, such as collagen, gelatin, lipids, and minerals, demonstrating the potentiality of this subject. The main goal of this review is to help researchers, policymakers and economic agents to understand the trends and the tools available to address such a relevant topic in the years to come.

## 7. Circular Economy in the Fishery Sector

The impact of fisheries on the environment is a hot topic, which is addressed at the global level by the FAO and the countries that pursue different strategies with the common goal to reach sustainable fisheries.

The way the fishery sector affects marine environment is multifold:

- Emissions: in 2011, the world fishing fleet burned 40 billion liters of fuels and emitted 179 million tons of CO_2_-equivalent (CO_2_-eq) of greenhouse gasses (GHG), corresponding to 2.2 kg of CO_2_-eq per kg of fish and invertebrates [161].
- Waste production: biological and non biological wastes produced during fisheries activities represent a growing concern [6];
- Overfishing: the intensification of primary production activities endangers the fish stocks and marine life that depends on them [162].

Most of these issues are generated by a diffuse and consolidated linear approach to production (“linear economy”) based on the “take-make-dispose” approach to natural resources.

The introduction of a circular economy perspective to fisheries activities is a strong driver to reverse the actual, not sustainable, trend of fishery sector towards a growing negative impact on aquatic environments.

The United Nations set up a general framework for the general strategy to reach fisheries sustainability [163], whereby they identified three pillars—economic development, social development and environmental protection—as essential to improve the sustainability indices of the sector. To fulfill this goal and cover all these aspects, the circular economy approach, based on a holistic perspective of the fishery sector, would provide general solutions: the reuse of waste would not only provide a solution for waste management, but also improve the economic performance of the whole sector and the social inclusion of all fishermen, turning into value also not edible products and sub-products.

According to the Ellen McArthur Foundation (https://www.ellenmacarthurfoundation.org/circular-economy/what-is-the-circular-economy) the circular economy approach relies on these founding elements:

- Ecodesign: each product and its production process must be designed to be further reused/recycled rather than disposed;
- Modularity and versatility: if a product is modular and versatile its use is strongly conservative again changing conditions (overcoming product obsolescence);
- Use of renewable energies: the use of renewable energy sources assures the sustainability of the process over time and non-renewable sources depletion;
- Ecosystem approach: the production processes must be designed accounting for all interactions with environment, so the products through “cradle-to-grave” environmental impact assessment;
- Materials recovery: the use of recycled materials must be recommended rather than virgin raw materials. In this framework, the use of fisheries by-catches or processing by-products, centrally falls in the strategic shift from the linear to the circular economy paradigm, providing elements to reduce the impact of fisheries sector, improving its economic performance.

### 7.1. The Biorefinery of Fisheries By-Catches or Processing By-products

The term "biorefinery" refers to the change of paradigm from the use of fossil sources (oil) for chemical industry to the use of renewable, biological sources (biomass). The main products of oil refineries are fuels, energy and chemicals: overcoming the oil era requires to build up an industrial analogue, providing the same products (energy, fuels and chemicals) out of biomass processing [164].

Biomasses include purposed crops, wastes or innovative biomasses fed on residual lands or ponds (algae). The use of wastes helps overcoming the “food vs. fuel” dilemma [165], endangering the development on a worldwide scale of bio-based chemical industry.

The biorefinery based on food discards support the food industry in solving the problem of the waste management, providing energy, fuels and chemicals from the chemical conversion of wastes [166]. Fishery discards include, fishery bycatches and fish processing industry by-products. The introduction of landing obligation (CFP Art. 15), poses in EU a stringent obligation on the landing of all catches of the fisheries activities [167]. This obligation, aimed at reducing the effect of the practice of discarding at sea of fished products with low or no commercial interest and the use of nonselective fisheries techniques, strongly challenged the fisheries sector in EU, facing the raising costs for landing products with no market placement possibilities [11,134]. 

Fish biorefinery represents an innovative approach to the fishery by-catch and processing by-products management [168]. Fishery discards are already considered by-products for the provision of products for human consumption (such as fish oil). At the same time, the use of fishery discards for energy production is under evaluation: the large humidity hinders the efficiency of energy production, but advanced techniques for biogas conversion seem to provide an efficient framework for energy conversion.

**Figure 1:**
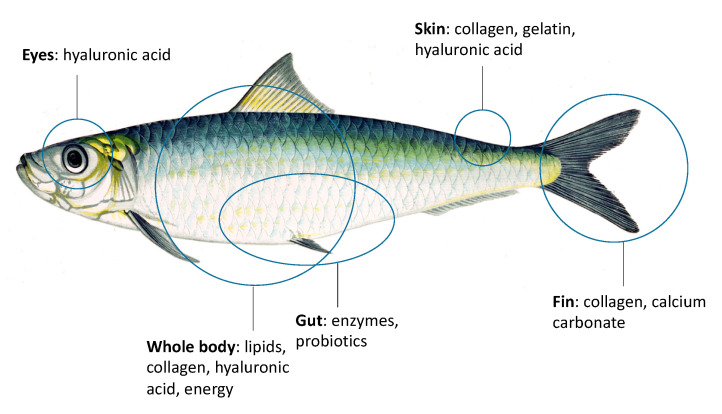
Added values from a fish body.

The biorefinery approach integrates all valorization routes for biomass in a single process, providing as outputs high value compounds, energy, fuels, and providing as well environmental services (fumes treatment and wastewater treatment). In this way, the processed biomass is completely converted into value (for instance, the energy conversion solid residual is converted into soil fertilizer). Figure 1 reports a scheme of the added values in a fish body as example of possible extraction routes for valorization of fishery discards.

### 7.2. Energy Production from Fishery By-Catch or Processing By-Products

The chemical energy of compounds which are present in the residual biomass (lipids, proteins and polysaccharides [169]), out of the separation stages for high value compounds, can be converted into thermal and electric energy. However, the high moisture content of fishery discards, and the presence of minerals require proper technologies for an efficient energy conversion.

The most reliable and simplest methodology for energy conversion of fishery discards (more in general, for wet biomasses) is biogas production [170]: the process is quite simple, but requires large volumes and occurs with low efficiency. The final biogas quality depends on the processed biomass and generally requires further processing (upgrading) to be converted into biomethane.

Residual fish oil, not proper for feed or human consumption, can be converted into biodiesel [158] or bio-oil [171]. This option allows also to delocalize the energy use into a liquid fuel, proper for transportation use as drop-in biofuel (i.e., that can replace traditional fuels from fossil sources without substantial changes of engines).

Finally, the use of torrefaction allows to use wet biomasses also in direct thermal conversion methods, which are characterized by high efficiency of energy conversion [172].

## 8. Conclusions

The production of large amounts of fishery by-catch and processing by-products and their disposal has severe environmental, economic and social implications. In this context, the valorization of fishery discards, so far considered of no economic value, plays a central role in solving the environmental impact of waste management. On the other hand, many studies report the large number of bioactive compounds contained in fishery by-catch and processing by-products, justifying the development of valorization routes for their extraction. While some fields of application of fishery by-catch/processing by-products are already consolidated (i.e., for aquafeeds), there are many applications that have been partially exploited to date and that stimulate future research (Table 6).

In a circular economy scenario, the use of exhausted effluents from the extraction stages for energy production would comply with the “zero waste strategy”, which is the new golden standard for the integrated supply chains. The use of energy produced by effluents conversion represents also a possible solution to decrease greenhouse gas emissions of the whole fisheries supply chain, a global concern to reduce the impact of fisheries activities in the scenario of climate change.

More recently, researchers are employing environmental-friendly extraction techniques that are able to extract the value-added molecules and to maintain their biological function. Several studies have been performed and value-added compounds have been successfully extracted from different fishery discards.

Collagen, enzymes, bioactive peptides, and gelatin were isolated from skin, scales, bones and muscles through enzymatic methodology. Polyunsaturated fatty acids (PUFAs) were obtained from fish residues using supercritical CO_2_ extraction and pressing, whereas hydroxyapatite was obtained by calcination and enzymatic methods from fish bones, frames, and scales. These wastes will serve to produce value-added products to satisfy the needs of the market. An exhaustive exploration of the fishery resources needs to be accomplished in a sustainable manner in the near future.

The single use of a natural resource to satisfy the needs of a single industry is not feasible, and the fish processing industry by-products could be a benchmark to fulfil the highly desired minimal waste/high throughput industry. Thus, the concept of a marine biorefinery has to be applied for a sensible exploitation of the available marine resources. A biorefinery is a multiple process and multiple product system wherein the residue coming out of a treatment process is used as feedstock for another process, where a maximum treatment efficiency is achieved, supporting the circular economy concept. Therefore, accounting also for the goal of zero waste, a crucial objective is to fully use the whole fish residues leaving a zero-carbon footprint instead of using some of fish processing by-products to produce few distinct products such as fish oil, collagen or gelatin. To this extent, and to the best of our knowledge, no studies were performed to implement a methodology to use all the fish substrate and leave no waste, to fully accomplish the zero-waste strategy. Moreover, further studies are encouraged to examine the bioactivity and bioavailability of protein peptides derived from fishery discards.

Extensive research on marine probiotics has been done and at present most of scientific studies described the action in vitro but less is known about the in vivo mechanisms (dose, time of administration, etc.) for a more efficient selection of potential probionts and to set up a “biological” model. Last but not least, it is important to determine any possible side effects on the environments since the addition of live cells can alter the microbial community and affect the turnover of organic and inorganic compounds in a given habitat [127]. Thus, interdisciplinary research is required among scientists to provide innovative approaches to investigate the marine sources, to identify, purify and to produce high amount of a specific bioactive compound, using modern technologies at low cost, in order to unlock the bioactive potential of marine microbes for biotechnological applications [97]. Further studies are necessary to understand the exact contribution of the gastrointestinal microbiota, because of the complexity and variable ecology of the digestive tract of different fish species [117], and from a scientific perspective we should have a better understanding of the mucosal–bacterial interactions, which mediate the host benefits, in order to achieve an optimal utilization in aquaculture. In synthesis, there are promising developments for fish probiotics and for the production of microbial biomolecules from fishery discards.

Research about the valorization of fishery discards is proliferating, with respect to technologies and processes used for the sustainable conversion of fishery resources to high added-value molecules and bio(nano)-materials [173]. From a practical point of view, for each processing technology the economic profitability must be taken into consideration: for example, regarding fish processing biorefinery, a specific analysis by Kratky and Zamazal [174] has shown that the methane yield and the methane purchase price are the two main factors affecting the profitability of this technology. A crucial role in the processing of fishery discards is also that related to the application of appropriate preservation technologies, therefore the availability of infrastructures and/or technologies close to the places of production of discards allows the drying operation and proper storage needed to guarantee their microbiological and physicochemical stability [175]. A waste-to-wealth concept, where waste valorization is considered for its intrinsic benefits to the environment as well as to develop new technologies, livelihoods and jobs, has been suggested by Xu et al. [176] as a way to promote a future sustainable lifestyle. Future studies will deal with nutritional, biotechnological and sensorial aspects, bioavailability of nutrients, interaction with other ingredients, technological and legislative issues, such as extraction methods and safety concerns. Fishery discards can give a significant contribution towards the economical transition and an optimal solution for fisheries-based industry, as well as a useful tool for a blue economy towards the zero-waste goal [177].

In conclusion, the use of fishery by-catch and processing by-products for nutritional and functional applications may represent a powerful tool in reducing the hunger of developing countries; moreover, the diversification of the productive chains can contribute to the creation of new job opportunities, with consequent important socio-economic and environmental benefits.

## Figures and Tables

**Table 1:** Main issues inherent the problem of fishery by-catch and processing by-products.

Fishery By-Catch and Processing By-Products		
Negative Issues	Positive Issues	
	Utilization of fishery by-products involves:	
Environmental impact	Contribution to fishery sustainability and environmentally friendly disposal methods	
Losses of fresh and high quality, potentially exploitable, bioactive compounds	Valorization of high-value compounds (i.e., probiotics, bioactive metabolites, enzymes, antibiotics)	New perspectives of application in nutritional, pharmaceutical, industrial sectors (i.e., biorefinery); beneficial effects for human and animal health (i.e., probiotics)
Lack of standardized protocols for extraction		
Operational costs for the use of fishery by-products	Green technologies allowing preservation and even enhancement of the quality and the extraction efficiency	

**Table 2:** Average composition of fish body (modified from Ghaly et al. [15]).

Component	Average Weight (%)
Fillet	36
Head	21
Bones	14
Fins	10
Gut	7
Liver	5
Skin	3
Ovaries	4

**Table 3:** Composition of fishery discards on a dry matter basis (modified from Ghaly et al. [17]).

Compound	Fish Discards
Crude protein (%)	57.92 ± 5.26
Ash (%)	21.79 ± 3.52
Fat (%)	19.10 ± 6.06
Crude fiber (%)	1.19 ± 1.21
Calcium (%)	5.80 ± 1.35
Phosphorous (%)	2.04 ± 0.64
Potassium (%)	0.68 ± 0.11
Sodium (%)	0.61 ± 0.08
Magnesium (%)	0.17 ± 0.04
Iron (mg/kg)	100.00 ± 42.00
Zinc (mg/kg)	62.00 ± 12.00
Manganese (mg/kg)	6.00 ± 7.00
Copper (mg/kg)	1.00 ± 1.00

**Table 4:** Content of high value components in fishery by-products (modified from Ferraro et al. [25]).

Fishery By-Products	High Value Components	Content (% *w/w*)	Market Value (Euro/kg)
Fish skin, scales and bones	Collagen and gelatin	Up to 80% in skin, up to 50% in scales	9–14
Fish skin, scales and bones	Hydroxyapatite	60–70% in bones, up to 50% in scales	not available
Fish viscera	Enzymes		14.400 (cod proteases)
White fish flesh residues	Free aminoacids	0.8–2% of taurin, 2.7% of creatine (on dry matter)	not available
Cod liver, mackerel oil	Polyunsaturated fatty acids-PUFA (ω3 and ω6)	50–80% in cod liver, 23% are w3 PUFA	24 (as cod liver oil)

**Table 5:** Representative studies on the potential probiotic activity of the microsymbionts isolated from fish organs of major economically important marine fish species.

Host Species	Fish Organs	Microbial Group/Species	Biomolecule/Type	Activity	References
*Scophthalmus maximus*, *Oncorhynchus mykiss*, *Hermosillaazurea*, *Dicentrarchus labrax*, *Anguilla japonica*, *Sparus aurata*, *Paralichthys olivaceus*	**gut**	**Bacteria** *Enterovibrio* spp., *Faecalibacterium*, *Desulfovibrio*, *Enterobacter* sp., *Aeromonas* spp., *Plesiomonas shigelloides*, *Hafnia alvei*, *Citrobacter freundii*, *Lysinibacillus fusiformis*, *Staphylococcus equorum*, *Flavobacterium sasangense*, *Shewanella xiamenensis*, *Vibrio pelagius*, *Vibrio* spp., *Photobacterium* sp., *Agrobacterium* sp., *Brevibacterium* sp., *Pseudomonas* spp., *Microbacterium* sp., *Staphylococcus* sp., *Acinetobacter* sp., *Sphingomonas* spp.	EPA (20:5 (n-3)), fatty acids (16:1 (n-7), 18:1 (n-9); 20:1, 22:1), SCFAs, acetate, propionate, valerate, butyrate, isobutyrate, formate, lactate, amilase, saccarase, cellulase, lipase; protease, chitinase, vitamin B_12_; glycolipid/rhamnolipid	Antimicrobial, antitumor, cardio-protective, immunomodulatory, digestive efficiency, energy supply, surfactant/bioemulsifying	[91,98,104,105,106,109,116,117]
*Chaeturichthys stigmatias*, *Salmo salar*, *Sparus aurata*, *Dicentrarchus labrax*, *Mugil cephalus*, *Gadus morhua*, *Lates calcarifer*, *Chanos chanos*, *Anguilla japonica*, *Epinephelus coioides*, *Solea solea*		*Carnobacterium* sp., *Enterococcus faecium*, *Lactobacillus pentosus*, *L. fructivorans*, *L. delbrueckii*, *Bacillus* spp., *B. cereus*, *B. subtilis*, *Brochothrix*, *Staphylococcus* sp., *Jeotgalibacillus* sp., *Psychrobacter* sp., *Leuconostoc* sp., *Micrococcus* spp., *Macrococcus* sp., *Microbacterium* sp., *Paenibacillus* spp.	Enterocin (peptide), intracellular, lactonase, amilase, cellulase, protease, phytase, lipase, chitinase	Antimicrobial, competitive exclusion of pathogens, immunomodulatory, growth promotion, fry mortality reduction, digestive efficiency	[88,90,94,100,101,102,103,112,113,114,115]
*Sparus aurata*, *Synaphobranchus kaupi*, *Oncorhynchus* spp., *Holothuria scabra*, *Hexagrammos otakii*, *Synecogobius hasts*, *Pleuronectes platessa*, *Scophthalmus maximus*, *Pagrus major*, *Platichthys flesus*, *Dicentrarchus labrax*, *Pomatomus saltatrix*		**Yeast** *Metschnikowia zobelii*, *Trichosporon cutaneum*, *Debaryomyces hansenii*, *Candida* spp., *Pichia* sp., *Kodamea ohmeri*, *Saccaromyces cerevisiae*, *Leucosporidium* sp., *Rhodotorula* spp.	Cell surface, glycoproteins, β-glucans, tannase, manno-proteins, chitin, phytase, extracellular proteases, siderophores, polyamines	Immunomodulatory, prebiotic, competitive exclusion of pathogens, growth promotion, feed efficiency, maturation of digestive system, increase larvae survival	[85,123,124,125]
*Hippoglossus hippoglossus*, *Chaeturichthys stigmatias*, *Salmo salar*, *Sparus aurata*, *Dicentrarchus labrax*, *Mugil cephalus*, *Lates calcarifer*, *Japanese eel*, *Epinephelus coioides*, *Solea solea*, *Oncorhynchus mykiss*, *Morone saxatilis*, *Scophthalmus maximus*	**gills/skin**	*Acinetobacter* sp., *Agrobacterium tumefaciens*, *Azospirillum orizae*, *Enterobacter* spp., *Erwinia persicina*, *Vibrio* spp., *Photobacterium* sp., *Pseudomonas* spp., *Moraxella* sp., *Sphingomonas* spp., *Myroides* spp., *Flavobacterium* spp., *Lactobacillus* spp., *Carnobacterium* sp., *Microccocus* spp., *Streptococcus* spp., *Kurthia* sp., *Clostridium* spp., Actinobacteria (*Arthrobacter arilaitensis*; *Mycobacterium* sp.), *Anoxybacillus* spp., *Bacillus cereus*, *Staphylococcus* sp., Cyanobacteria, **Fungi** (*Aspergillus* spp., *Candida santamariae*, *Trichosporon laibachii*), **Yeast**	Peptides; (N/I)	Antimicrobial, immunomodulatory, increased fish and larvae survival, competitive esclusion of pathogens	[85,126,127,128,129,131]

(N/I): not identified.

**Table 6:** Current and future perspectives of application of fishery by-catch/processing by-products.

Conventional	Novel and in Progress
Ingredients animal/fish feed (fishmeal, fish oil, fish sauces, fish silage) [17]	Pharmaceutical and industrial, ingredients for nutraceutical, cosmetic, agricultural fields (bioactive peptides [33,34,35,36,40], FPH [44], hyaluronic acid [108])
Nutritional supplements, food industry, pharmaceutical (EPA–DHA, lipids, minerals) [13,25]	Low-cost systems for managing and valorizing fishery discards, industrial and biomedical applications (enzymes [66], collagen, gelatin [70,71])
	Pharmaceutical (cancer drugs) and food industry (chitin and chitosan) [75,77,78]
	Aquaculture, biomedical, food industry (probiotics), bioremediation [85,98,99,118,119,120,126,127,128,129]
	Energy production (biodiesel or bio-oil production from fish oil) [156,157,158,159,160,161,162,163,164,165,166,167,168,169,170]
